# Intestinal ultrasound in primary intestinal lymphangiectasia (Waldmann’s disease): a PRISMA-ScR scoping review with an illustrative adult case

**DOI:** 10.1007/s40477-026-01181-5

**Published:** 2026-07-29

**Authors:** Luisa Bertin, Miriana Zanconato, Camilla Cavagna, Brigida Barberio, Cristina Caranfil, Cristina Bezzio, Patrizia Burra, Edoardo Vincenzo Savarino, Fabiana Zingone

**Affiliations:** 1 Gastroenterology Unit, Azienda Ospedale Università Padova, Via Giustiniani 2, 35128 Padua, Italy; 2 Department of Surgery, Oncology and Gastroenterology (DiSCOG), University of Padova, Padua, Italy; 3 IBD Center, IRCCS Humanitas Research Hospital, Rozzano, Milan, Italy; 4 Department of Biomedical Sciences, Humanitas University, Pieve Emanuele, Milan, Italy

**Keywords:** Primary intestinal lymphangiectasia, Waldmann’s disease, Intestinal ultrasound, Bowel wall thickening, Mesenteric lymphangiectasia, Protein-losing enteropathy, Scoping review, Hennekam syndrome, Colour Doppler, Limberg score

## Abstract

Primary intestinal lymphangiectasia (PIL; Waldmann’s disease, ORPHA 90362) is a rare protein-losing enteropathy in which dilated intestinal lacteals leak chyle into the gut lumen. Cross-sectional imaging and lymphoscintigraphy have historically dominated assessment, while transabdominal B-mode intestinal ultrasound (IUS)—a cornerstone modality for inflammatory bowel disease—has remained almost unmapped in PIL. We conducted a PRISMA-ScR scoping review (Open Science Framework preregistration gfbc9) of all primary studies reporting transabdominal B-mode IUS in PIL, searching three bibliographic platforms (PubMed/MEDLINE, Ovid Multifile, Scopus) supplemented by forward and backward citation chasing. Studies using only extra-intestinal ultrasound, endoscopic ultrasound, those describing secondary lymphangiectasia or focal lymphangioma, and those using ultrasound only as procedural guidance for lymphangiography were excluded at full-text stage. Fifteen primary studies, published 1986–2026 across thirteen countries, met eligibility (nine paediatric, six adult). All fifteen reported per-patient B-mode IUS findings extractable for synthesis. A consistent sonographic signature emerged: diffuse, regular, slightly hypoechoic small-bowel-wall thickening with preserved five-layer stratification and prominent valvulae conniventes; dilated fluid-filled loops with reduced peristalsis; oedematous mesentery, variable ascites; and characteristically absent mesenteric lymphadenopathy (with two notable exceptions framed only as a research hypothesis). Diagnostic accuracy as a screening tool has been evaluated only in one study (*n* = 20: accuracy 80%, 95% CI 56.3–94.3%). We illustrate this signature in a 42-year-old woman with Hennekam syndrome and report quantitative B-mode IUS values as anecdotal single-observation—single sonographer, single device, single fasted time-point, mid-jejunal segment to motivate, prospective standardisation.

**Supplementary Information:**

The online version contains supplementary material available at https://doi.org/10.1007/s40477-026-01181-5.

## Introduction

Intestinal ultrasound (IUS) has transitioned from a niche radiological technique to a recommended bedside modality for the diagnosis and longitudinal monitoring of inflammatory bowel disease, where pooled data and successive European and international guidelines have established its reproducibility, accuracy and responsiveness to treatment [1–6]. As IUS proliferates outside specialist radiology suites and into routine gastroenterology practice, the clinician increasingly encounters bowel-wall thickening in patients without inflammatory bowel disease. The differential then broadens to a heterogeneous group of rarer enteropathies, several of which can mimic Crohn’s disease sonographically yet require radically different management.

Primary intestinal lymphangiectasia (PIL), originally described by Waldmann and colleagues in 1961 and subsequently characterised by Strober [7–9], is one such mimic. It is a rare disorder of the intestinal lymphatic system (ORPHA 90362; estimated prevalence < 1/1,000,000 [7–9]) in which obstruction of small-bowel lymphatic drainage causes secondary dilation of mucosal, submucosal and subserosal lacteals; chyle leaks into the gut lumen, producing the cardinal triad of hypoalbuminaemia, lymphocytopenia and peripheral oedema. PIL is most often diagnosed before three years of age but adult-onset and very-late-onset cases are well described [10]. It may occur as an isolated finding or within multisystem genetic syndromes—most notably Hennekam lymphangiectasia–lymphoedema syndrome, caused by biallelic variants in CCBE1, FAT4 or ADAMTS3 [11,12]. Diagnosis rests on the combination of compatible biochemistry, raised faecal α1-antitrypsin clearance, the endoscopic ‘snowflake’ appearance of the duodenal mucosa, and confirmatory histology of dilated villous lacteals [13]. Imaging has so far focused on cross-sectional modalities—CT enterography, MR enterography and MR lymphography—and on functional studies such as 99mTc-albumin scintigraphy [14,15].

Despite its non-invasive, radiation-free, repeatable and inexpensive nature, IUS has scarcely been mapped in PIL. The European Federation of Societies for Ultrasound in Medicine and Biology gastrointestinal-ultrasound task-force consensus on rare gastrointestinal disorders touches only briefly on PIL [16], and the most recent French national diagnostic and care protocol does not yet position IUS within its diagnostic pathway [17]. To our knowledge, no synthesis of IUS findings in PIL has been published.

We therefore performed a PRISMA-ScR scoping review of primary studies reporting transabdominal IUS in PIL, with the following objectives: (i) to chart the full extent, range and characteristics of the IUS evidence in PIL; (ii) to synthesise a sonographic signature and a structured differential diagnosis; and (iii) to illustrate this signature with the IUS measurements and Colour Doppler data in an adult Hennekam-syndrome case. The intended audiences are gastroenterologists, paediatricians and radiologists who increasingly perform IUS at the bedside and may encounter PIL among the unexplained causes of bowel-wall thickening.

## Methods

### Protocol and registration

This scoping review was conducted in accordance with the JBI methodology for scoping reviews [18] and reported following the PRISMA-ScR (Preferred Reporting Items for Systematic reviews and Meta-Analyses extension for Scoping Reviews) checklist [19]. The protocol was preregistered prospectively on the Open Science Framework (identifier gfbc9) before extraction began. The review question was: “What is the extent, range and characteristics of the published evidence on transabdominal intestinal ultrasound in patients with primary intestinal lymphangiectasia?”.

### Eligibility criteria

Eligibility criteria followed the Population–Concept–Context (PCC) framework.

Population: humans of any age with primary intestinal lymphangiectasia, including syndromic forms (Hennekam, Noonan, Turner, Klippel–Trénaunay, tuberous sclerosis complex), but excluding secondary intestinal lymphangiectasia from heart failure, pericardial disease, lymphoma, infiltrative disorders or post-radiation; we also excluded papers that, on full-text reading, described focal solitary small-bowel lymphangioma rather than the diffuse protein-losing PIL phenotype.

Concept: transabdominal B-mode intestinal ultrasound of the small or large bowel and surrounding mesentery, defined operationally as a deliberate dedicated examination of the bowel wall (thickness, stratification, valvulae, mesentery) using high-frequency linear or convex transducers consistent with the EFSUMB-IBUS framework, as distinct from generic abdominal ultrasonography in which bowel walls were not separately assessed. Studies whose only ultrasound was extra-intestinal (cardiac transthoracic, parathyroid, peripheral lymphoedema), studies whose modality was endoscopic ultrasound, studies whose only sonographic data were Doppler interrogation of mesenteric vessels without any concurrent B-mode bowel-wall assessment, and studies in which ultrasound was used only as procedural guidance for intra-nodal or trans-hepatic lymphangiography rather than for bowel-wall imaging were all excluded a priori as outside the B-mode IUS concept. Klingenberg 2003 was retained as an included study because, although its principal sonographic dataset was duplex-Doppler tracking of mesenteric arteries during slow-release-octreotide treatment, the same examination also documented an extractable B-mode bowel-wall finding ("large amounts of free abdominal fluid and oedematous thickening of small intestinal walls") that meets the inclusion criterion [20]. Doppler IUS, contrast-enhanced ultrasound, intestinal elastography and three-dimensional IUS were intentionally not part of the included concept and are addressed only in the Discussion as adjacent modalities of future research interest.

Context: any clinical setting (paediatric, adult, antenatal, peri-operative, emergency, outpatient) and any country. Source types eligible: original primary studies—single case reports, case series, prospective and retrospective cohorts, and diagnostic-accuracy studies. Reviews, editorials, pictorial essays, conference abstracts, textbook chapters and first-person patient narratives without primary IUS data were used only as background references and were not included in the synthesis. No language, year or publication-status restrictions were applied; non-English full texts (Chinese, French, Spanish) were assessed with native-speaker support and verified machine translation.

### Information sources and search strategy

We searched three bibliographic platforms from inception to the final search date (10 May 2026): MEDLINE/PubMed, an Ovid Multifile cross-search combining Ovid Embase, Ovid MEDLINE(R) and the All-EBM Reviews aggregate (which includes the Cochrane Database of Systematic Reviews and CENTRAL), and Scopus (Elsevier). PubMed returned 137 records, the Ovid Multifile 968 (after platform-level deduplication of overlapping MEDLINE/Embase/Cochrane hits), and Scopus 480, for a combined database-stream yield of 1585 records. After deduplication of these database records in EndNote 21 and Rayyan (564 within-database-stream duplicates removed), 1021 unique database records remained for title-abstract screening. A further 14 records were identified through forward and backward citation chasing on every included primary study and on prior narrative reviews of PIL imaging; these were carried forward through the parallel "identification of new studies via other methods" arm of the PRISMA-2020 flow and assessed for eligibility separately. Across the two streams, the total number of records identified was 1599 (1585 database plus 14 citation-chased). The complete electronic search strings, including all wildcards, MeSH terms and field tags, are reported in Supplementary Material 1, the PRISMA-ScR checklist is presented as Supplementary Material 2. Two authors independently assessed retrieved titles and abstracts; full-text assessment was carried out independently by the same two reviewers; disagreements were resolved by discussion with a senior reviewer.

### Selection, data charting and critical appraisal

A standardised charting form, piloted on five randomly chosen records and refined iteratively, was used by two authors independently in duplicate to extract bibliographic metadata, study design and sample characteristics; the IUS protocol (transducer frequency; presence of Doppler, contrast or elastography); and per-patient IUS findings (bowel-wall thickness in millimetres, loop diameter in centimetres, wall stratification, valvulae, peristalsis, mesenteric appearance, lymphadenopathy, ascites and Doppler signal). Studies in which IUS was mentioned without per-patient B-mode bowel-wall findings extractable from the available text were excluded from synthesis. Methodological quality was appraised descriptively using the Murad et al. tool [21] across its four domains; per-study results are reported in Supplementary Material 3 and the aggregate counts are summarised in the Results. We did not exclude reports based on appraisal, in keeping with the scoping nature of the review.

### Synthesis of results

Data were synthesised narratively and tabulated. Where multiple studies reported the same descriptive feature (e.g. presence or absence of ascites), proportions were calculated with simple counts and denominators; no formal meta-analysis was attempted, in line with PRISMA-ScR recommendations and the heterogeneity of the source studies. The diagnostic-accuracy estimates from the single eligible accuracy study (Zhang 2020 [22]) are reported verbatim and not pooled. Studies were grouped by age category (paediatric ≤ 18 years vs adult or older adult), by IUS modality (B-mode only vs B-mode plus Doppler vs advanced techniques), and by IUS yield (typical signature, atypical/segmental, normal/non-diagnostic). Disagreements between the two synthesisers were resolved by consensus.

### Illustrative case

To anchor the synthesis in current IUS practice, we report a single illustrative adult case examined at the authors' institution. The patient gave written informed consent for publication. Ethics approval and consent for the single illustrative case were processed under the institutional case-report framework, with full text deferred to the Declarations on the title page.

## Results

### Search results and selection

**Fig. 1:**
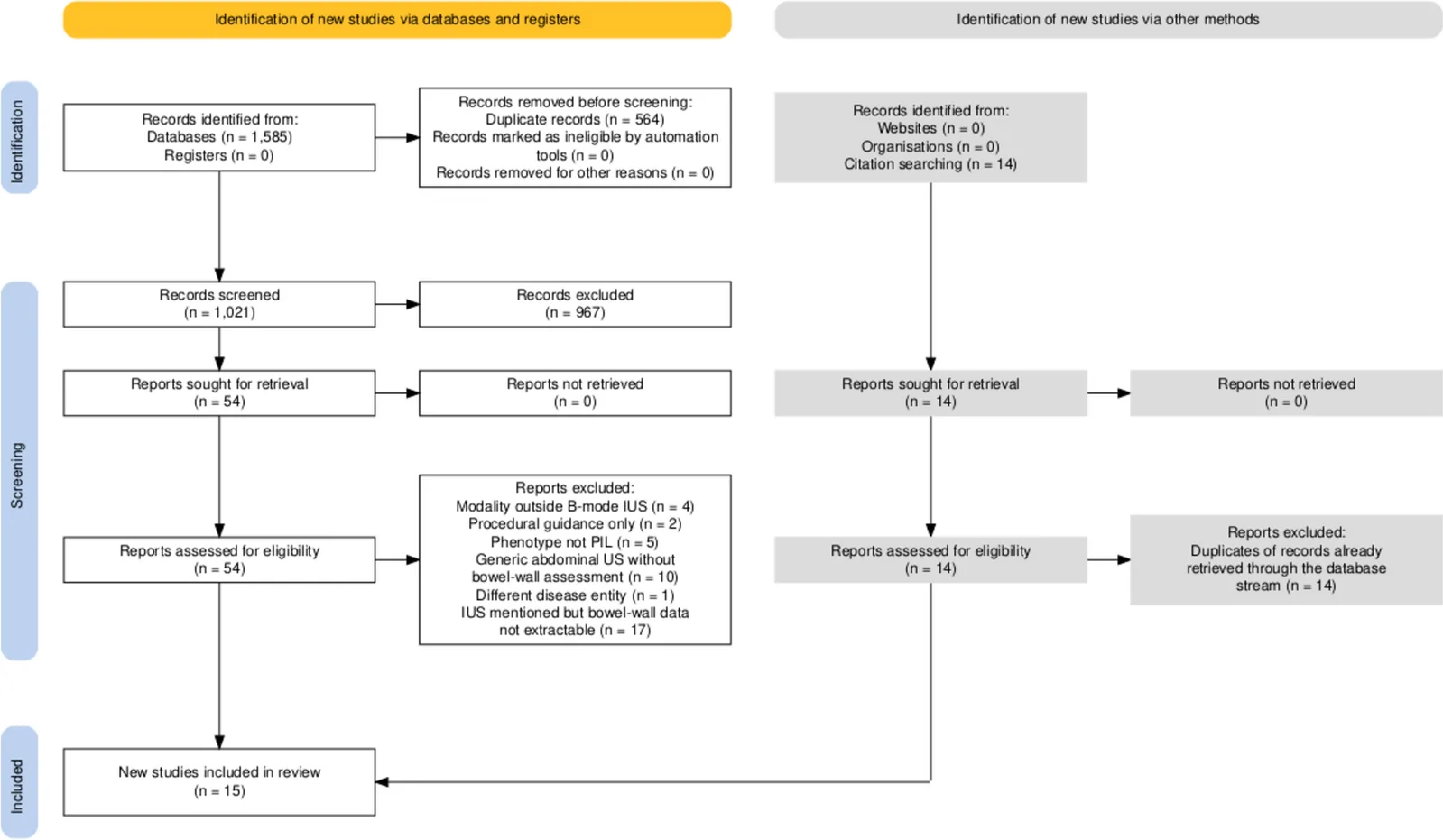
PRISMA-ScR study selection flow diagram [44]

The PRISMA-ScR study selection process is summarised in Fig. 1, drawn per the PRISMA-2020 "databases and other methods" template with the two streams shown in parallel. Database searching identified 1585 records (137 PubMed, 968 Ovid Multifile, 480 Scopus); after within-database-stream deduplication (564 duplicates removed), 1021 unique database records advanced to title-abstract screening. Of these, 967 were excluded at the title-abstract stage and 54 were sought for retrieval and assessed for full-text eligibility (Cohen’s κ for the title-abstract phase = 0.82). In parallel, 14 records were identified through forward and backward citation chasing; all 14 were sought for retrieval and assessed for full-text eligibility, and all 14 were found to be duplicates of records already retrieved through the database stream, contributing no net new inclusions. The total number of records identified across the two streams was therefore 1599 (1585 database plus 14 citation-chased). Thirty-nine full-text records were excluded with reasons grouped by category: modality outside B-mode IUS (*n* = 4); ultrasound used only as procedural guidance for intra-nodal or trans-hepatic lymphangiography (*n* = 2); phenotype not PIL (*n* = 5); generic abdominal ultrasonography without dedicated bowel-wall assessment (*n* = 10), different disease entity (*n* = 1, Ménétrier protein-losing gastropathy); and intestinal ultrasound mentioned without extractable per-patient B-mode bowel-wall findings from the available text (*n* = 17). Fifteen primary studies, published between 1986 and 2026 across thirteen countries, met the eligibility criteria and were included in the scoping review.

### Characteristics of the included studies

Of the fifteen included studies, nine included paediatric and six adult patients for a total of 44 subjects. The included studies originate from thirteen countries, listed alphabetically: Bahrain, Canada, China (multiple centres including Hunan, Shanghai), Germany, India, Italy, Japan, Morocco, Nepal, Portugal, South Korea, Syria and Taiwan. All fifteen included studies provided per-patient B-mode IUS findings extractable for synthesis (Table 1). All included studies relied on B-mode imaging with linear and convex transducers (typically 3.5–7.5 MHz convex and 7.5–12 MHz linear); three studies (Klingenberg 2003, Alshikho 2016 and Pais-Cunha 2024) reported ancillary Colour or duplex Doppler interrogation in addition to a B-mode bowel-wall assessment [20, 23,24]; no included study reported contrast-enhanced ultrasound, intestinal elastography or three-dimensional IUS in PIL.

**Table 1:** Overview of the 15 primary studies included in the B-mode IUS-focused scoping review of primary intestinal lymphangiectasia

#	Author (year)	n	Setting	Country	Salient IUS finding
1	Dorne (1986) [26]	3	Paediatric	Canada	First IUS series; ascites + diffuse bowel-wall thickening + mesenteric oedema + dilated lymphatics; gallbladder/bladder-wall thickening
2	Maconi (1998) [7]	1	Adult	Italy	First systematic adult IUS protocol: regular hypoechoic wall thickening, prominent valvulae, dilated lymphatics in oedematous mesentery, mild ascites
3	Klingenberg (2003) [20]	1	Adult	Germany	Combined B-mode and duplex Doppler: large amounts of free abdominal fluid, oedematous thickening of small intestinal walls; serial mesenteric-artery Doppler tracked octreotide response
4	Loreti (2003) [25]	1	Adult	Italy	Enteroclysis + sonography in a 26-year-old female with malabsorption; B-mode US showed mural thickening of small-bowel loops; diagnosis confirmed at biopsy
5	Oh (2011) [27]	1	Adult	South Korea	Diffuse hypoechoic wall thickening, dilated loops, prominent folds, mild ascites at admission; capsule endoscopy and double-balloon enteroscopy followed
6	Isa (2016) [42]	2	Paediatric	Bahrain	Patient 1: unremarkable abdominal US; Patient 2: initial US read as normal but retrospective review revealed halo sign of thickened intestinal wall
7	Alshikho (2016) [24]	1	Adult	Syria	B-mode IUS in the diagnostic workup; ancillary Colour Doppler reported
8	Li S (2017) [28]	9	Paediatric	China	Abdominal B-mode US showed varying degrees of oedema and effusion in all nine infants; serial monitoring during nutritional therapy
9	Zhang (2020) [22]	20	Paediatric	China	Only diagnostic-accuracy study: accuracy 80%, sensitivity 92.31%, specificity 57.14%, PPV 80%, NPV 80%
10	El Haddar (2021) [29]	1	Paediatric	Morocco	Abdominal US showed a peritoneal effusion of great volume; trans-thoracic US ruled out cardiac causes; small-bowel wall thickening normalised after octreotide
11	Prajapati (2022) [30]	1	Paediatric	Nepal	Whole-abdomen US: moderate ascites, multiple enlarged mesenteric lymph nodes, mildly diffuse mucosal thickening of the bowel loop; normal liver/spleen/kidney
12	Pais-Cunha (2024) [23]	1	Paediatric	Portugal	B-mode IUS for diagnosis (intermittent ileo-ileal intussusception, moderate ascites), exclusion of cardiac/portal cause and treatment monitoring; ancillary Doppler reported
13	Sekine (2024) [31]	1	Adult	Japan	Transabdominal US: massive ascites, enlarged celiac lymph nodes, and oedematous thickening of small intestinal wall
14	Chen (2025) [32]	1	Paediatric	Taiwan	Diffuse bowel-loop thickening and ascites prompted paracentesis (chylous, TG 1093 mg/dL); follow-up US showed resolution of ascites and improvement of bowel-wall oedema after dietary therapy
15	Gaur (2026) [33]	1	Paediatric	India	Equivocal/non-diagnostic US due to bowel gas and overlapping loops in PIL with intussusception

All entries (sorted by year) provided per-patient B-mode IUS findings extractable for the synthesis tables

### Synthesis of sonographic findings

Across the fifteen included studies, a recurrent IUS pattern was apparent. Diffuse, regular, slightly hypoechoic small-bowel wall thickening with preserved five-layer stratification was the dominant grayscale finding [7, 20, 23–32]. Loop dilation with increased intraluminal anechoic fluid was reported uniformly when explicitly assessed [7, 26, 28,29]; valvulae conniventes were described as prominent or thickened in the published images and text [7, 26]. Mesenteric oedema with discrete dilated lymphatic channels—a feature first illustrated by Maconi 1998 in an adult patient with long-standing disease—was repeatedly observed [7, 24–26]. Mesenteric lymphadenopathy was characteristically absent in the original adult IUS descriptions [7, 25]; however, two reports document enlarged mesenteric or coeliac lymph nodes in PIL—Prajapati 2022 (paediatric, Nepal) [30] and Sekine 2024 (adult, Japan) [31]— challenging the long-held dogma that lymph-node absence is universal in PIL. Ascites was variable in extent: explicitly present in three of three children examined by Dorne and Jequier and in adult cases with severe protein-losing enteropathy, but mild or absent in less advanced disease [7, 20, 25,26, 28,29, 32]. The first published serial-monitoring data come from Chen 2025, in whom a follow-up ultrasonogram showed resolution of ascites and improvement of bowel-wall oedema after initiation of dietary therapy [32] (see Table 2).

**Table 2:** Side-by-side comparison of the three landmark per-patient IUS descriptions in primary intestinal lymphangiectasia (Dorne 1986, Maconi 1998 and the present case 2025)

Feature	Dorne 1986 (*n* = 3, paed.)	Maconi 1998 (*n* = 1, adult)	Present case 2025 (*n* = 1, adult)
Patient age	Paediatric (newborn–13 y)	20 y, female	42 y, female
Disease distribution	Diffuse	Diffuse	Diffuse
Bowel-wall thickening	Diffuse, grossly thickened	Slightly hypoechoic, regular, diffuse	Slightly hypoechoic, regular, diffuse
Wall thickness (mm)	Not reported	Not reported	3.3
Intestinal folds	Thickened	Prominent valvulae	Prominent valvulae conniventes
Loop dilation	Present	Jejunal & ileal dilation	Jejunal & ileal dilation
Loop diameter (cm)	Not reported	Not reported	3.68
Mesentery	Marked oedema, dilated lymphatics	Markedly oedematous, dilated lymphatics	Markedly oedematous, dilated lymphatics
Lymphadenopathy	Absent	Absent	Absent
Ascites	Present (3/3)	Mild	Mild
Other organ involvement	Gallbladder + bladder-wall thickening	None reported	None
Clinical presentation	Typical PIL	Recurrent sub-occlusion	Sub-occlusive episodes; chronic PIL

The present case provides the first published quantitative IUS values

### Diagnostic accuracy and IUS yield

Only one study has formally evaluated the diagnostic accuracy of IUS in PIL: Zhang 2020, a Chinese-language paediatric series of twenty children with suspected PIL who underwent high-frequency abdominal ultrasound and concurrent reference-standard endoscopy with biopsy [22]. The reported overall accuracy was 80% (16/20; exact binomial 95% CI 56.3–94.3%), with sensitivity 92.31% (12/13; 95% CI 64.0–99.8%), specificity 57.14% (4/7; 95% CI 18.4–90.1%), positive predictive value 80% (12/15; 95% CI 51.9–95.7%) and negative predictive value 80% (4/5; 95% CI 28.4–99.5%). The wide specificity confidence interval—with a lower bound of 18.4%, well below chance—reflects the small denominator of seven non-PIL controls and tempers the authors' own conclusion that IUS is suitable as a first-line screening tool: with seven controls the lower bound of the specificity estimate cannot exclude that IUS distinguishes PIL from non-PIL no better than chance, even though the point estimate is 57.1%. A further methodological caveat applies: the histological reference standard (endoscopic biopsy with confirmation of dilated lacteals) was available in 13 of 20 study participants; the seven non-PIL participants were classified as non-PIL on the basis of clinical and biochemical work-up rather than histology, so the specificity calculation rests on a different reference standard for the seven controls than for the thirteen PIL cases. The 80% accuracy figure should therefore be interpreted with corresponding caution. No equivalent diagnostic-accuracy study has been published in adult or syndromic PIL. One report documents negative or non-diagnostic abdominal ultrasound in patients subsequently confirmed to have PIL: Gaur 2026 (paediatric, India, with intussusception and IUS deemed equivocal because of bowel gas) [33]. More studies are needed before considering IUS as a screening protocol for patients with suspected PIL.

### B-mode and advanced ultrasound modalities

Beyond conventional B-mode imaging, no included study used contrast-enhanced ultrasound, no study used intestinal elastography, and no study used three-dimensional IUS in PIL. Three included studies reported ancillary Colour or duplex Doppler interrogation in addition to a B-mode bowel-wall assessment without using Doppler as a stand-alone diagnostic modality (Klingenberg 2003, Alshikho 2016 and Pais-Cunha 2024) [20, 23,24]. Klingenberg 2003 is the central methodological precedent for prospective Doppler-IUS, CEUS and elastography work in PIL: in a 27-year-old woman with type I lymphangiectasia, the same examination showed B-mode evidence of ‘large amounts of free abdominal fluid and oedematous thickening of small intestinal gut walls’ and serial duplex tracking of slow-release-octreotide pharmacodynamics in the superior and inferior mesenteric arteries [20].

### Illustrative adult case

To illustrate the IUS signature synthesised above, we report a single adult case examined at the authors' institution.

### Patient and prior workup

A 42-year-old woman with Hennekam lymphangiectasia–lymphoedema syndrome (compound heterozygous CCBE1 variants confirmed genetically) was referred for IUS evaluation in the context of recurrent sub-occlusive episodes and chronic protein-losing enteropathy. The patient had a long-standing history of bilateral lower-limb lymphoedema, recurrent chylous effusions, hypoalbuminaemia and lymphocytopenia. Imaging-guided workup at the local centre had included video capsule endoscopy at age 24; the disease was clinically stable on dietary management with intermittent supportive hospitalisation through the following decade, with imaging deferred until renewed sub-occlusive symptoms prompted MR enterography at age 39; colonoscopy with biopsy followed at age 40. Genetic, dietetic and clinical management had been multidisciplinary; medication included supplemental medium-chain triglycerides and intermittent octreotide.

### Intestinal ultrasound technique

The IUS examination was performed by a single experienced sonographer using a GE LOGIQ S8 system with a high-frequency linear transducer (L3-12, 9.0 MHz) for B-mode bowel-wall assessment and a curvilinear transducer (C1-6, 4.0 MHz) for wider abdominal survey, after a four-hour fast in supine and lateral decubitus positions. The abdomen was scanned in a systematic ‘lawnmower’ pattern with dedicated longitudinal and transverse views of duodenum, jejunum and ileum. Bowel-wall thickness was measured perpendicular to the wall on a longitudinal section of a non-collapsed proximal jejunal loop (mean of three measurements); loop diameter was measured outer-to-outer on a transverse section of the most dilated jejunal segment. An exploratory Colour Doppler interrogation graded on the modified Limberg scale was also performed. On the day of the IUS examination the patient had been fasting for four hours per the standard protocol; she was clinically stable, with no acute sub-occlusive episode in the preceding two weeks, and her last subcutaneous octreotide dose had been administered on the previous evening, so the IUS findings reflect a steady-state interictal-and-treated condition rather than an acute decompensation.

### Sonographic findings

**Fig. 2:**
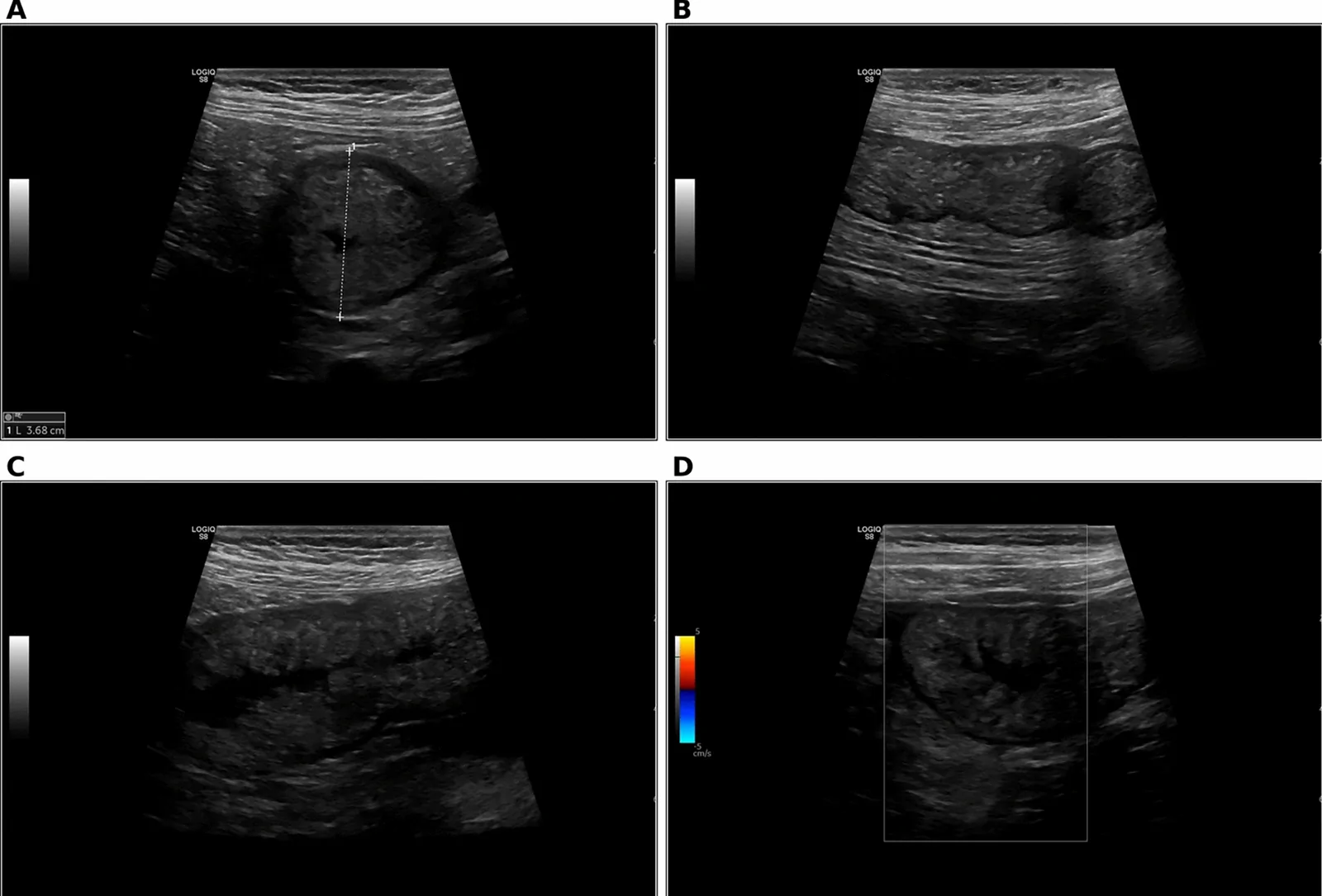
Intestinal ultrasound findings in primary intestinal lymphangiectasia. **A** Longitudinal and transversal ultrasonographic section of an intestinal loop with hypoechoic, regular thickened bowel wall, along with prominent valvulae and dilated lymphatic vessel. Loop diameter measures 3.68 cm. **B** A transverse section of a dilated loop with increased intraluminal fluid and ascites. **C** Hypertrophic and oedematous mesentery with dilated lymphatic vessels and dilated intestinal loops. **D** Color Doppler examination of the affected bowel segment. All images obtained using GE LOGIQ S8 with L3-12 linear transducer at 9.0 MHz

**Fig. 3:**
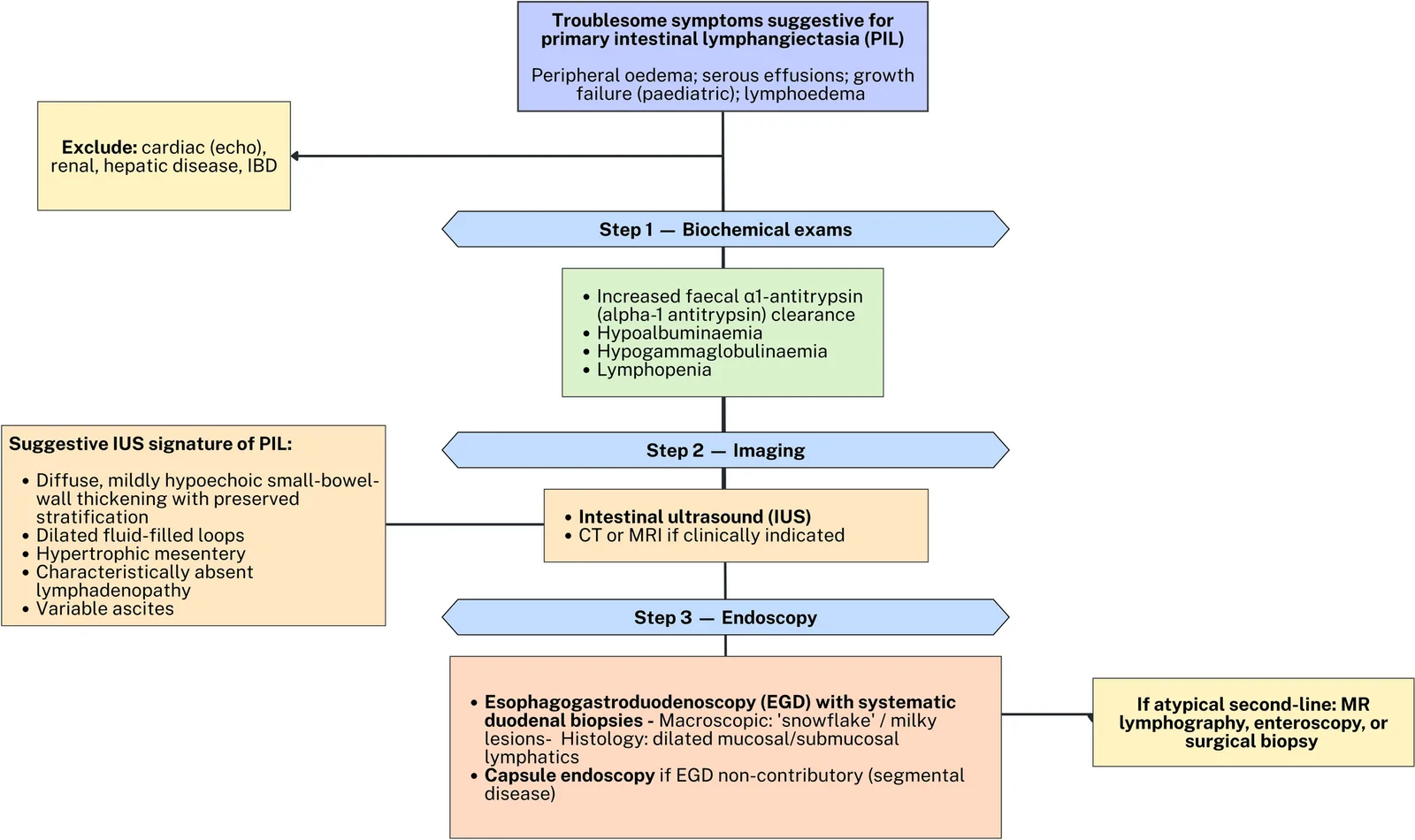
Proposed diagnostic algorithm for the IUS evaluation of suspected primary intestinal lymphangiectasia

Sonography demonstrated the full PIL signature inferred from the literature synthesis (Fig. 2). Diffuse, regular, slightly hypoechoic small-bowel-wall thickening was visible in the jejunum and ileum, with preserved five-layer stratification and prominent valvulae conniventes. Mean bowel-wall thickness measured 3.3 mm in this single examination—only marginally above the 3 mm IBD-derived threshold for inflammatory bowel-wall thickening [16], and within measurement-error of normality (an *n* = 1 anecdotal value, single sonographer, single device, single fasted time-point, mid-jejunal segment). The most dilated jejunal loop measured 3.68 cm in transverse outer-to-outer diameter (normal values up to 2.5 cm [34]), with anechoic intraluminal fluid and reduced peristaltic activity. The mesentery was diffusely hypertrophic. Mild ascites was visible in the peri-hepatic and inter-loop spaces. No mesenteric, retroperitoneal or coeliac lymphadenopathy was detected. The exploratory Colour Doppler interrogation of the bowel wall revealed only a single small intramural focus of vascular signal, corresponding to modified Limberg score 1 in a segment, while most segments had a corresponding modified Limberg score of 0 (see Fig. 3).

### Synthesised IUS signature and differential diagnosis

Integrating the fourteen published per-patient B-mode datasets with the present case, the IUS signature of PIL can be summarised as five co-occurring features: diffuse, regular, slightly hypoechoic small-bowel-wall thickening (typically 3–6 mm) with preserved five-layer stratification; dilated fluid-filled loops with prominent valvulae conniventes and reduced peristalsis; hypertrophic mesentery; absent mesenteric lymphadenopathy in the great majority of reports, with the documented exceptions in Prajapati 2022 and Sekine 2024 [30,31]; and variable ascites. The first three features are constant; absent lymphadenopathy is highly characteristic but not universal; ascites tracks with severity. This signature must be distinguished from active Crohn’s disease, intestinal lymphoma, intestinal amyloidosis, coeliac disease (including the cavitating lymph-node syndrome of refractory disease), systemic sclerosis, eosinophilic gastroenteritis and Whipple disease; rare paediatric mimics include Noonan-syndrome diffuse intestinal lymphangiectasia and congenital vascular malformations of the bowel [35,36]. A structured side-by-side comparison of IUS features across these differential entities is provided in Table 3.

**Table 3:** Differential diagnosis of diffuse small-bowel-wall thickening on intestinal ultrasound: principal IUS features in primary intestinal lymphangiectasia versus six common or important mimics

IUS feature	PIL	Crohn	Lymphoma	Coeliac	Systemic sclerosis	Eosinophilic gastroenteritis	Amyloidosis
Wall thickness	Mild–moderate (3–6 mm)	Marked (> 3 mm; often 5–10 mm)	Marked, pseudo-cystic	Normal–mild (moderate in refractory)	Normal–mild	Mild–moderate (mucosal)	Mild–moderate
Distribution	Diffuse, symmetric	Segmental, skip	Focal/segmental	Diffuse (jej > ileum)	Diffuse (duod/jej)	Diffuse (gastric + SB)	Diffuse
Stratification	Preserved (5 layers)	Lost	Lost	Often preserved	Preserved	Preserved	Preserved/blurred
Doppler / Limberg	Minimal in single illustrative case (Limberg 0–1, *n* = 1 anecdotal observation)	Increased (2–4)	Variable	Variable	Absent	Variable	Hypovascular
Lymphadenopathy	Absent (rare exception)	May be present	Present (key)	Often present (small, reactive); cavitating LN in refractory CD	Absent	Occasional	Present
Intraluminal fluid	Increased	Variable	Variable	Increased	Stagnant (retained)	Frequent (mild)	Variable
Loop dilation	Diffuse	Pre-stenotic	Variable	Mild	Marked (hidebound)	Moderate	Variable
Mesentery	Oedematous, dilated lymphatics	Creeping fat, comb sign	Mass-like nodes	Reactive LN; cavitating in refractory	Normal	Ascites (serosal)	Dilated vessels, nodes
Peristalsis	Reduced	Variable	Variable	Increased	Markedly reduced	Variable	Reduced
Key distinguishing sign	Absent nodes + dilated lymphatics	Stenosis + comb sign	Target/bull’s-eye sign	Reversed jejuno-ileal pattern + reactive nodes	Hidebound + diverticula	Piano-key + eosinophilia	Nodes + napkin-ring

Cell-level features synthesised from the EFSUMB GIUS Task Force consensus on celiac sprue and rare gastrointestinal disorders [16], the Cavalcoli 2017 review of small-bowel ultrasound beyond IBD [43], the included primary literature for the PIL column

## Discussion

This PRISMA-ScR scoping review and accompanying illustrative case together represent, to our knowledge, the first systematic synthesis dedicated to transabdominal B-mode intestinal ultrasound in primary intestinal lymphangiectasia. The EFSUMB Gastrointestinal Ultrasound Task Force position paper on celiac sprue and rare gastrointestinal disorders [16] briefly addresses PIL within a broader catalogue; the present work expands and updates that mapping with a dedicated, preregistered, multilingual search and a per-patient synthesis. The synthesis of fifteen primary studies published over four decades and thirteen countries reveals a strikingly consistent IUS signature that has hitherto been articulated only piecemeal in single case reports, separated by language barriers and by the rarity of the disease. The signature—diffuse, regular, slightly hypoechoic small-bowel-wall thickening with preserved five-layer stratification, dilated fluid-filled loops with prominent valvulae conniventes, hypertrophic mesentery, characteristically absent lymphadenopathy and variable ascites—is internally coherent across age groups and anatomical territories, and consistent with the lymphatic, non-inflammatory pathophysiology of the disease. The illustrative adult Hennekam-syndrome case documents this signature with quantitative B-mode IUS values offered, to our knowledge for the first time in the included literature, as a starting reference for prospective standardisation.

A central finding of the review is that IUS sensitivity in PIL, although high in the only formal accuracy study (Zhang 2020 reported 92.3% in paediatric subjects), is not absolute [22], although evidence comes from one study only and should therefore be interpreted with caution, particularly in the adult setting.

A more provocative observation concerns mesenteric lymphadenopathy. The original adult IUS descriptions of Dorne 1986 and Maconi 1998 explicitly emphasise the absence of enlarged lymph nodes in PIL, and this absence has since been propagated as a near-universal differentiating feature from lymphoma and other infiltrative mimics [7, 26] Two reports—Prajapati 2022 in a paediatric Nepalese patient (multiple enlarged mesenteric lymph nodes with mildly diffuse mucosal thickening on whole-abdomen ultrasonography) and Sekine 2024 in an adult Japanese patient (massive ascites, enlarged celiac lymph nodes, and edematous thickening of the small intestinal wall on transabdominal ultrasonography)—document enlarged coeliac or mesenteric lymph nodes in unequivocally diagnosed PIL [30,31]. With only two outlier reports out of fifteen included studies, this observation is statistically very limited and cannot in itself displace the long-held universal-absence dogma. Several alternative explanations for the lymphadenopathy in these two cases cannot be excluded on the available data, including concurrent inflammation, reactive (rather than malignant or PIL-specific) nodes, sampling differences between sonographers and operators, and—in Sekine 2024 specifically—the very advanced disease state with massive ascites and possibly secondary lymphatic congestion at the time of imaging. We therefore frame this finding strictly as a hypothesis-generating observation worth raising for prospective multicentre study, rather than as actionable clinical guidance; a denominator of dozens to hundreds of cases followed for decades would be needed to test it formally. Given that intestinal B-cell lymphoma has been reported in roughly 5% of PIL cases with long-standing disease, with an average latency of three decades [37], whether IUS-detected lymphadenopathy could in future serve as an early sonographic alert for malignant transformation is, on these data, an open question that this scoping review can frame but cannot answer; multicentre prospective data are needed before any change in surveillance practice would be justified.

Our synthesis identifies the variables that prospective multicentre studies of IUS in PIL should capture in a standardised manner: bowel-wall thickness measured perpendicular to the wall in millimetres in matched jejunal and ileal segments; layer stratification graded as preserved or lost; loop diameter in centimetres on transverse outer-to-outer measurement; the prominence of valvulae conniventes and qualitative peristalsis; mesenteric oedema and the presence and short-axis size of dilated lymphatic channels and lymph nodes; the depth of ascites in the peri-hepatic, peri-splenic, pelvic and inter-loop compartments; and the intramural Limberg-grade Doppler signal. Anchoring future PIL imaging research in the EFSUMB GIUS framework [3, 16, 38] and the variable set used for IBD treat-to-target monitoring [4] would enable cross-disease comparability. The development of a fully specified standardised IUS protocol for PIL, including image-archive standards and follow-up cadence, is, in our view, a logical companion paper that should itself be preregistered and would benefit from international rare-disease network endorsement; it lies beyond the scope of the present scoping review.

The treatment-monitoring potential of B-mode IUS in PIL is, on the basis of this review, almost entirely unexploited; Doppler IUS, contrast-enhanced ultrasound and intestinal elastography are clearly identified future directions rather than current practice. Klingenberg 2003 remains the only published combined B-mode-and-Doppler study in PIL and provides the methodological precedent for prospective Doppler-IUS work [20]. In IBD, serial B-mode IUS—including bowel-wall thickness, intramural Doppler grading and the IBUS-SAS activity score—has become the modality of choice for treat-to-target monitoring [39]. Transferring the B-mode component of this paradigm to PIL is plausible: the four quantitative parameters proposed here (bowel-wall thickness, loop diameter, mesentery score, ascites depth) are obtainable at the bedside, reproducible in trained hands, and physiologically responsive to the dietary, pharmacological or surgical interventions that constitute the current armamentarium [17]. Chen 2025 provides initial proof of bedside responsiveness, with serial IUS documenting resolution of ascites and improvement of bowel-wall oedema after dietary therapy [32]. The Limberg-score-1 observation in the present case is reported as a single anecdotal data point to motivate, not pre-empt, formal prospective study. B-mode IUS is also radiation-free, contrast-free and repeatable at the bedside, with a substantially lower environmental footprint than CT, MRI or endoscopy (0.5–1.5 kg CO₂-equivalent per scan vs 17–22 kg for MRI, 7–10 kg for CT and 7.8–56.4 kg per endoscopy procedure [40])— a growing concern for lifelong monitoring of a chronic rare disease.

Several limitations should temper interpretation. First, despite a comprehensive search only one diagnostic-accuracy study exists in the entire literature, and it is paediatric, single-centre, Chinese-language and modest in size; the evidence base in adults remains entirely composed of case reports and small series, with all of the methodological concerns that this entails. Second, although the IUS signature is internally consistent, it is not pathognomonic: amyloidosis, diffuse Noonan-syndrome lymphangiectasia and rare vascular malformations can produce overlapping pictures, and an unexpected segmental or appendicitis-mimicking presentation is possible (described in case reports of localised/focal PIL excluded from the present synthesis) [41]. Third, no included study used contrast-enhanced ultrasound, intestinal elastography or three-dimensional IUS in PIL—modalities that may, in future, refine the differential by quantifying perfusion and stiffness. Fourth, this scoping review by design did not pool data, did not formally meta-analyse and did not exclude reports for methodological quality; we used the Murad case-report appraisal tool descriptively only. Fifth, our review necessarily inherited the publication and language biases of the source literature, although the inclusion of Chinese-language, French-language and Spanish-language reports mitigates this in part. Finally, the illustrative case is, by definition, a single observation; its quantitative values must be interpreted as a starting reference rather than a normative benchmark, and prospective multicentre validation is the obvious next step.

## Conclusions

Fifteen primary studies, spanning four decades and thirteen countries, show that IUS in PIL produces a coherent and internally consistent sonographic signature: diffuse, regular, slightly hypoechoic bowel-wall thickening with preserved stratification, dilated fluid-filled loops with prominent valvulae, oedematous mesentery with dilated lymphatic channels, characteristically absent mesenteric lymphadenopathy with rare documented exceptions, and variable ascites. Translating these findings into clinical practice will require prospective multicentre validation through European and international rare-disease networks. Doppler IUS, contrast-enhanced ultrasound and intestinal elastography are framed as future research directions rather than current practice. Such validation is, in our view, the single most important next step needed to position IUS within the diagnostic and monitoring pathway of primary intestinal lymphangiectasia.

## Supplementary Information

Below is the link to the electronic supplementary material.

Supplementary file1 (DOCX 19 KB)

Supplementary file2 (DOCX 46 KB)

Supplementary file3 (DOCX 28 KB)

## Data Availability

All data analysed during this scoping review are derived from publicly available primary publications cited in the reference list. The screening database, charting form, full search history, and de-identified images of the illustrative case are available from the corresponding author on reasonable request, in line with the Open Science Framework preregistration (identifier gfbc9).

## Declarations

### Use of large language models

Anthropic’s Claude was used for AI-assisted language refinement. All conceptualisation, data extraction, methodological decisions, scientific interpretation and final wording are the authors’ own; no LLM contribution meets the JUS authorship criteria. The authors are accountable for the final version of the text.
