# Supplementary material for: Intestinal ultrasound in primary intestinal lymphangiectasia (Waldmann’s disease): a PRISMA-ScR scoping review with an illustrative adult case
Source: J Ultrasound. 2026 Jul 29;29(4):949–61. doi: 10.1007/s40477-026-01181-5 (PMC13768459; doi:10.1007/s40477-026-01181-5)
Supplement: Supplementary file 1 [file 40477_2026_1181_MOESM1_ESM.docx]

**Supplementary Material 1**

**Search strategy**

This supplementary material reproduces the search strategy used for this scoping review of intestinal ultrasound in primary intestinal lymphangiectasia. The review was conducted according to the JBI Manual for Evidence Synthesis (Peters MDJ et al., 2024 update) and reported according to the PRISMA Extension for Scoping Reviews (Tricco AC et al., Ann Intern Med 2018;169:467–473). The protocol was registered prospectively on the Open Science Framework before the formal database searches were performed (OSF identifier gfbc9; DOI to be inserted at acceptance).

## **1. Population, Concept, Context (PCC)**

### **Population**

Human patients of any age (neonatal, paediatric, adolescent, adult, geriatric) with confirmed or strongly suspected primary intestinal lymphangiectasia (PIL; Waldmann’s disease; ORPHA code 90362), irrespective of underlying genetic syndrome (Hennekam, Noonan, Klippel–Trénaunay, von Recklinghausen, Turner, tuberous sclerosis complex) when the lymphangiectasia is the primary digestive lesion.

### **Concept**

Any abdominal application of ultrasonography, including transabdominal B-mode, intestinal ultrasound (IUS) or sonography of the small bowel, high-frequency bowel-wall sonography, colour and spectral Doppler of mesenteric vessels, point-of-care ultrasound (POCUS), small-bowel contrast ultrasonography (SICUS), contrast-enhanced ultrasound (CEUS), strain or shear-wave elastography, and endoscopic ultrasound (EUS). Cardiac and obstetric ultrasound are excluded except when the cardiac or fetal study is the only sonographic examination performed and is essential to interpreting the case.

### **Context**

Any healthcare setting, any country, any year. No language restrictions are applied. Animal studies, fetal-only studies without postnatal follow-up, and secondary intestinal lymphangiectasia (cardiac, hepatic, infiltrative, post-surgical, malrotation-associated, infectious, drug-induced) are excluded.

## **2. Inclusion and exclusion criteria**

### **Inclusion**

Human subjects with primary intestinal lymphangiectasia in whom abdominal ultrasonography (any of the modalities listed above) was performed and reported. Any study design including case reports, case series, retrospective and prospective cohorts, cross-sectional studies, registry analyses, narrative and systematic reviews, conference abstracts, theses, and clinical practice guidelines.

### **Exclusion**

Secondary lymphangiectasia, fetal-only series with no postnatal follow-up, studies in which intestinal ultrasound was not performed or not reported, animal studies, duplicates and republications, and editorials and letters that do not contain primary data.

## **3. Databases searched**

Three bibliographic databases were searched from inception to the final search date of 10 May 2026. The PubMed (NLM/MEDLINE) interface yielded 137 records. A single Ovid Multifile cross-search combining Ovid Embase, Ovid MEDLINE(R) and the All EBM Reviews aggregate yielded 968 records after platform deduplication. Scopus (Elsevier) yielded 480 records. The combined database-stream yield was therefore 1,585 records (137 + 968 + 480). Within-database-stream deduplication in EndNote 21 and Rayyan removed 564 duplicates, leaving 1,021 unique database records that advanced to title-abstract screening. A further 14 records were identified through forward and backward citation chasing on every included primary study and on prior narrative reviews of PIL imaging; these 14 records were carried forward through the parallel "identification of new studies via other methods" arm of the PRISMA-2020 flow rather than added to the database screening pool. Across the two streams, the total number of records identified was 1,599 (1,585 database plus 14 citation-chased). Each of the 14 citation-chased records was then sought for retrieval, retrieved and assessed for full-text eligibility in parallel with the 54 database-stream reports that advanced from title-abstract screening; on full-text assessment, all 14 citation-chased records were found to be duplicates of records already retrieved through the database stream after deduplication, and the citation-chasing arm therefore contributed no net new inclusions to the synthesis. The corresponding numbers in the PRISMA-2020 / PRISMA-ScR flow diagram (Figure 1) are: 1,585 database records identified, 564 within-stream duplicates removed, 1,021 unique database records screened, 967 excluded at title-abstract stage, 54 reports sought and 54 assessed, 39 excluded with reasons and 15 included (database stream); 14 records identified, 14 reports sought, 14 assessed and 14 excluded as duplicates of database-stream records (citation-chasing stream); 15 studies included in the scoping review (15 from databases plus 0 net new from citation chasing).

Grey literature was searched on Google Scholar (first 200 results screened), ProQuest Dissertations and Theses Global, ClinicalTrials.gov, the WHO ICTRP, and the abstract repositories of UEG Week, Digestive Disease Week (DDW), ESPGHAN, NASPGHAN, the International Bowel Ultrasound Group (IBUS) annual course, RSNA and ECR. Reference lists of all included studies and of the principal PIL practice guidelines (Vignes & Bellanger 2008; Vignes et al. PNDS 2025; Kwon & Kim 2021) were hand-searched. Forward citation tracking was performed in Scopus on the seminal sonographic papers (Dorne 1986; Maconi 1998; Klingenberg 2003; Malone 2015; Zhang 2020).

## **4. Search strings as run**

### **4.1 PubMed (MEDLINE) — 137 records retrieved**

```
("Lymphangiectasis, Intestinal"[Mesh]
  OR "intestinal lymphangiectasia"[tiab]
  OR "primary intestinal lymphangiectasia"[tiab]
  OR "Waldmann disease"[tiab]
  OR "Waldmann's disease"[tiab]
  OR "Waldmanns disease"[tiab]
  OR "protein-losing enteropathy"[tiab]
  OR "protein losing enteropathy"[tiab])
AND
("Ultrasonography"[Mesh]
  OR ultraso*[tiab]
  OR sonograph*[tiab]
  OR echograph*[tiab]
  OR doppler[tiab]
  OR CEUS[tiab]
  OR "contrast enhanced ultrasound"[tiab]
  OR "intestinal ultrasound"[tiab]
  OR "bowel ultrasound"[tiab]
  OR POCUS[tiab])
```

### **4.2 Ovid Multifile (combined Ovid resources accessed via the institutional Ovid platform) — 968 records retrieved**

The Ovid Multifile search combined the following resources, all selected on the Ovid resource picker on the search date and visible in the institutional saved search history. Coverage windows are reproduced verbatim from the Ovid platform display.

```
• EBM Reviews — ACP Journal Club  (1991 to April 2026)
• EBM Reviews — Cochrane Central Register of Controlled Trials  (March 2026)
• EBM Reviews — Cochrane Database of Systematic Reviews  (2005 to April 30, 2026)
• EBM Reviews — Cochrane Clinical Answers  (April 2026)
• EBM Reviews — Cochrane Methodology Register  (3rd Quarter 2012)
• EBM Reviews — Database of Abstracts of Reviews of Effects  (1st Quarter 2016)
• EBM Reviews — Health Technology Assessment  (4th Quarter 2016)
• Embase  (1974 to 2026 Week 18)
• Ovid MEDLINE(R) and Epub Ahead of Print, In-Process, In-Data-Review & Other Non-Indexed Citations and Daily  (1946 to May 08, 2026)
```

Search strategy as run on the combined set:

1. exp intestinal lymphangiectasia/

2. (intestinal lymphangiectasia or primary intestinal lymphangiectasia or Waldmann*).ti,ab,kw.

3. exp protein losing enteropathy/

4. ("protein losing enteropathy" or "protein-losing enteropathy").ti,ab,kw.

5. 1 or 2 or 3 or 4

6. exp echography/

7. exp ultrasound/

8. (ultraso* or sonograph* or echograph* or doppler or CEUS or POCUS or "intestinal ultrasound" or "bowel ultrasound").ti,ab,kw.

9. 6 or 7 or 8

10. 5 and 9

Records retrieved (combined): 968

### **4.3 Scopus (Elsevier) — 480 records retrieved**

```
TITLE-ABS-KEY ( "intestinal lymphangiectasia" OR "primary intestinal lymphangiectasia"
                OR "Waldmann disease" OR "Waldmann’s disease"
                OR "Waldmanns disease" OR "protein-losing enteropathy"
                OR "protein losing enteropathy" )
AND TITLE-ABS-KEY ( ultraso* OR sonograph* OR echograph* OR doppler
                OR CEUS OR POCUS OR "contrast enhanced ultrasound"
                OR "intestinal ultrasound" OR "bowel ultrasound" )
```

The three database-stream electronic sets (PubMed, Ovid Multifile, Scopus, total 1,585 records) were merged in EndNote 21 and Rayyan and deduplicated, with 564 within-database-stream duplicates removed, leaving 1,021 unique database records. These 1,021 unique records were loaded into Rayyan for title-abstract screening. The 14 citation-chased records, separately curated, were carried forward through the parallel "identification of new studies via other methods" arm of the PRISMA-2020 flow and assessed in parallel for full-text eligibility. Numbers identified, after deduplication, screened, sought for retrieval, assessed for eligibility, excluded with reasons and finally included are reported, separately for each stream, in the PRISMA-ScR flow diagram (Figure 1).

## **5. Sensitivity validation against a known-item list**

Sensitivity was validated against a pre-specified known-item list of 50 PIL plus US studies identified during scoping and listed in the supplementary inventory accompanying this protocol (PIL_IUS_scoping_review_inventory.xlsx). Records expected to be retrievable in PubMed and Ovid were checked individually against the 968-record Ovid set. Any expected paper not retrieved represented a sensitivity gap and prompted refinement before formal screening. One inventory item was not retrievable through the three database platforms and was addressed by other means: the AGW 2022 Bhana conference abstract, captured by hand-search of the GESA/AGW supplement. Loreti L et al. 2003, initially flagged during scoping as a potential abstract-only citation, was subsequently retrieved in full from the Ovid Multifile set on the formal search date and is included in the synthesis. Citation chasing on the seminal sonographic papers (Dorne 1986, Maconi 1998, Klingenberg 2003 and Zhang 2020) was performed in parallel as a sensitivity check; all 14 records identified in this way were found, on full-text assessment, to be duplicates of records already retrieved through the database stream after deduplication, indicating that the three-database search achieved adequate coverage of the available IUS-in-PIL literature.

## **6. Translation, deduplication and screening**

Records were exported in RIS format and managed in EndNote 21 and Rayyan. Deduplication was performed first automatically in EndNote, then manually in Rayyan. Two reviewers independently screened titles and abstracts, then full texts, with disagreements resolved by a third reviewer. Inter-rater agreement (Cohen’s κ) for the title-abstract phase is reported in the main manuscript Methods and Figure 1. Translation of non-English-language full texts (French, Italian, Spanish, Portuguese, Chinese, Japanese, Korean) was performed by native-speaker collaborators where possible, otherwise by a hybrid pipeline of DeepL Pro followed by manual physician review. Each translated extraction was cross-verified by a second reviewer against the original text.

## **7. Data charting**

A standardised charting form, piloted on five included studies and refined iteratively, was used. Fields mirror the columns of the supplementary inventory (PIL_IUS_scoping_review_inventory.xlsx) and include bibliographic metadata, country, study design, sample size, age and sex distribution, inclusion and diagnostic criteria for PIL, ultrasonographic modality and protocol, ultrasonographic findings (bowel-wall thickness in millimetres where reported, layer stratification, vascularity score, mesenteric fat changes, lymph-node enlargement, ascites, valvulae prominence, dilated lymphatic vessels), role of US in the diagnostic and follow-up pathway, comparator imaging, histology, treatment, response and outcome.

## **8. Risk of bias and synthesis**

Because this is a scoping review, formal risk-of-bias assessment is not required by the JBI methodology. The methodological quality of the included case reports and case series was nonetheless appraised with the Murad et al. tool (BMJ Evid Based Med 2018), and for the only larger cohort in the included synthesis (Zhang 2020, n = 20) with the JBI critical appraisal checklist for case series. Per-study results are reported in Supplementary Material 3. Synthesis is narrative and thematic, organised by (i) age group (paediatric vs adult), (ii) ultrasonographic modality, and (iii) clinical context (diagnostic vs treatment-monitoring vs complication-detection). No meta-analysis is performed because the evidence base consists predominantly of single-patient case reports and no two studies share an extractable diagnostic-accuracy outcome.

## **9. Limitations stated a priori**

The dominant evidence base is single-case reports without per-modality breakdown. Only one diagnostic-accuracy study exists (Zhang 2020, n = 20 paediatric, single centre, retrospective, with histological reference standard in only 13/20 patients), and no equivalent has been performed in adults or with modern multimodality IUS protocols (Doppler, CEUS, SICUS, elastography). Four papers identified during full-text screening (Díaz-Gómez 2024; Goret 2026 n = 34; Na 2024 n = 12; Mitsiakos 2018) reported aggregate or context-only ultrasonographic findings without extractable per-patient B-mode bowel-wall data and were excluded from the synthesis at full-text stage for that reason. Goret 2026 and Na 2024, the two largest available cohorts, reported aggregate radiological findings combining ultrasonography with cross-sectional imaging without per-modality breakdown; corresponding-author contact to obtain per-patient ultrasound data was attempted without success during the present scoping review.

## **10. Date of last search and reproducibility**

All electronic searches were last run on 10 May 2026, the search date visible on the Ovid platform display screenshots archived in the institutional records of the authors. The search history files (PubMed export, Ovid history) are available on request, in line with PRISMA-ScR item 7 and Open Science Framework preregistration gfbc9.
