# Supplementary material for: Intestinal ultrasound in primary intestinal lymphangiectasia (Waldmann’s disease): a PRISMA-ScR scoping review with an illustrative adult case
Source: J Ultrasound. 2026 Jul 29;29(4):949–61. doi: 10.1007/s40477-026-01181-5 (PMC13768459; doi:10.1007/s40477-026-01181-5)
Supplement: Supplementary file 3 [file 40477_2026_1181_MOESM3_ESM.docx]

**Supplementary Material 3**

**Murad descriptive methodological appraisal of the 15 included primary studies**

This descriptive appraisal applies the four domains of the Murad et al. tool for the methodological quality of case reports and case series (BMJ Evid Based Med 2018;23:60–63) to each of the 15 primary studies included in the present scoping review. Following the scoping-review methodology, no study was excluded on the basis of this appraisal; the table is provided for transparency and to allow readers to weigh which contributions to the synthesised IUS signature came from higher- or lower-quality sources. Domain definitions follow Murad et al.: Selection — does the case represent the whole experience of the investigator, or is the selection method unclear; Ascertainment — was the exposure (PIL diagnosis) adequately ascertained; Causality — is the causal link between exposure and outcome plausible, with sufficient follow-up; Reporting — is the case described with sufficient detail.

**Aggregate summary**

Across the 15 included studies, ascertainment of the PIL diagnosis was adequate in all 15 (diagnosis confirmed by duodenal/jejunal endoscopic biopsy with histological evidence of dilated lacteals, supplemented by raised faecal α₁-antitrypsin clearance where reported). Reporting of B-mode IUS findings was adequate in 14 of 15, with the single exception of Zhang 2020, in which the full diagnostic-accuracy data are reported only in the Chinese-language original and parts of the per-patient breakdown could not be extracted from the available English-language abstract. Selection was adequate (consecutive or prospectively enrolled cases over a defined window) in 4 of the 14 — Dorne 1986, Isa 2016, Li S 2017 and Zhang 2020; the remaining 11 are single-patient case reports in which selection by design is not separately assessable. Causality (sufficient follow-up to assess outcome) was adequate in 14 of 15, with the single exception of Zhang 2020 (cross-sectional diagnostic-accuracy design without per-patient outcome follow-up). These appraisal characteristics are typical of the rare-disease IUS literature and are appropriately accommodated by a scoping rather than a systematic review methodology.

**Per-study appraisal**

**Codes:** + adequate per Murad domain definition; ? unclear from the report as published; − not adequate per Murad domain definition. n.a. — not applicable (single case report; selection by design).

| # | Author (year) | n | Selection | Ascertainment | Causality | Reporting | Notes |
| --- | --- | --- | --- | --- | --- | --- | --- |
| 1 | Dorne (1986) | 3 | + | + | + | + | Three consecutive paediatric cases |
| 2 | Maconi (1998) | 1 | n.a. | + | + | + | Foundational adult IUS protocol |
| 3 | Klingenberg (2003) | 1 | n.a. | + | + | + | Combined B-mode and duplex Doppler; serial follow-up |
| 4 | Loreti (2003) | 1 | n.a. | + | + | + | Adult case report; B-mode US mural thickening of small-bowel loops; biopsy-confirmed |
| 5 | Oh (2011) | 1 | n.a. | + | + | + | Multi-modality workup |
| 6 | Isa (2016) | 2 | + | + | + | + | Two-patient paediatric series |
| 7 | Alshikho (2016) | 1 | n.a. | + | + | + | B-mode + Colour Doppler |
| 8 | Li S (2017) | 9 | + | + | + | + | Largest infant series with serial monitoring |
| 9 | Zhang (2020) | 20 | + | + | ? | ? | Diagnostic-accuracy study; full data not available in English |
| 10 | El Haddar (2021) | 1 | n.a. | + | + | + | Adolescent paediatric case |
| 11 | Prajapati (2022) | 1 | n.a. | + | + | + | Lymphadenopathy outlier |
| 12 | Pais-Cunha (2024) | 1 | n.a. | + | + | + | Paediatric; B-mode + Doppler |
| 13 | Sekine (2024) | 1 | n.a. | + | + | + | Adult lymphadenopathy outlier; Denver shunt follow-up |
| 14 | Chen (2025) | 1 | n.a. | + | + | + | Serial-monitoring proof of bedside responsiveness |
| 15 | Gaur (2026) | 1 | n.a. | + | + | + | Equivocal-IUS sensitivity caveat |

In summary, ascertainment of the PIL diagnosis was adequate in all 15 included studies. Reporting fidelity for B-mode IUS findings was adequate in 14 of 15, with Zhang 2020 the single exception because the full diagnostic-accuracy data are reported only in the Chinese-language original. Selection was adequate (consecutive or prospective enrolment) in 4 of the 15; the remaining 11 are single-patient case reports in which selection by design is not separately assessable. Causality (sufficient follow-up to assess outcome) was adequate in 14 of 15. These appraisal characteristics are typical of the rare-disease IUS literature and are appropriately accommodated by a scoping rather than a systematic review methodology.
